# Antibiotic use and risk of colorectal cancer: a systematic review and dose–response meta-analysis

**DOI:** 10.1038/s41416-020-01082-2

**Published:** 2020-09-24

**Authors:** Johanna Simin, Romina Fornes, Qing Liu, Renate Slind Olsen, Steven Callens, Lars Engstrand, Nele Brusselaers

**Affiliations:** 1 Centre for Translational Microbiome Research (CTMR), Department of Microbiology, Tumor and Cell Biology, Karolinska Institutet, Biomedicum kvarter 8A. Solnavägen 9, SE-171 65 Stockholm, Sweden; 2 Science for Life Laboratory (SciLifeLab), SE-171 21, Stockholm, Sweden; 3 Department of Clinical Genetics, Karolinska University Hospital, SE-171 76 Stockholm, Sweden; 4 Department of Internal Medicine, Ghent University Hospital, Ghent, Belgium

**Keywords:** Oncology, Diseases, Medical research, Cancer

## Abstract

**Background:**

It is understudied whether the posed association of oral antibiotics with colorectal cancer (CRC) varies between antibiotic spectrums, colorectal continuum, and if a non-linear dose-dependent relationship is present.

**Design:**

Three electronic databases and a trial platform were searched for all relevant studies, from inception until February 2020, without restrictions. Random-effects meta-analyses provided pooled effect-sizes (ES) with 95% confidence intervals (CI). Dose–response analyses modelling the relationship between number of days exposed to antibiotics and CRC risk were extended to non-linear multivariable random-effects models.

**Results:**

Of 6483 identified publications ten were eligible, including 4.1 million individuals and over 73,550 CRC cases. The pooled CRC risk was increased among individuals who ever-used antibiotics (ES = 1.17, 95%CI 1.05–1.30), particularly for broad-spectrum antibiotics (ES = 1.70, 95%CI 1.26–2.30), but not for narrow-spectrum antibiotic (ES = 1.11, 95% 0.93–1.32). The dose–response analysis did not provide strong evidence of any particular dose–response association, and the risk patterns were rather similar for colon and rectal cancer.

**Discussion:**

The antibiotic use associated CRC risk seemingly differs between broad- and narrow-spectrum antibiotics, and possibly within the colorectal continuum. It remains unclear whether this association is causal, requiring more mechanistic studies and further clarification of drug–microbiome interactions.

## Background

Colorectal cancer (CRC) is the fourth most common cause of death globally,^1^ accounting for ten percent of all incident cancers and cancer-related deaths annually.^1,2^ While the incidence is declining in several high-income countries,^3^ a yet unexplained increase among younger individuals (<50 years) has been observed in several continents,^3–5^ making this an important global health problem. Gut microbes have been associated with CRC promotion, particularly certain strains of *Escherichia coli* and *Fusobacterium nucleatum*. The latter might even involve a worse prognosis, and higher treatment resistance, if expressed at high levels.^6–8^

Antibiotics have been posed as a risk factor for CRC. Besides the global burden of antibiotic resistance, the high annual antibiotic consumption (20–50% globally)^4,9,10^ highlights the need for better understanding of the possible role of antibiotics and gut microbiome on the risk of CRC. Emerging evidence supports the hypothesis of oral antibiotics changing the gut microbiota composition,^11–13^ dysregulating critical host immune responses, and potentially changing important functions of the gut microbiome.^10,11,14,15^ These effects on the gut microbiome may be strong and persistent, possibly leading to chronic inflammation and tumour progression of even more distal tumour locations.^8–10,15–17^ However, the association of antibiotics with CRC is complex. Infection is another major risk factor for cancer globally, contributing to approximately 16% of annual incident cancers.^18,19^ Some evidence indicates that anti-inflammatory drugs may reduce the risk of cancer,^20^ further supporting the role of inflammation in carcinogenesis. Thus, it is also possible that antibiotics could reduce the risk of cancer, by decreasing inflammation.

Previous meta-analyses have suffered from power limitations including only five to six studies in total,^16,21^ pooling together colon adenoma with carcinoma^22^, or combining the different anatomical locations.^16,21^ None of them considered departure from linearity, although a recent study suggested for a non-linear dose–response relationship between antibiotic use and CRC risk.^9^ There is a clear lack of solid evidence summarising the potential association of antibiotics with CRC, and current evidence is insufficient to evaluate whether the potential CRC risk may differ within the colorectal continuum, or by broad- and narrow-spectrum antibiotics, indication, age or sex.^16,21,22^ With the aim of filling these knowledge gaps, we conducted this comprehensive systematic review and dose–response meta-analysis, evaluating the risk of CRC among individuals who ever-used antibiotics.

## Methods

This systematic review and meta-analysis was based on an a priori established study protocol. The results are reported in line with the Preferred Reporting Items for Systematic Reviews and Meta-analyses (PRISMA) guidelines.^23^

### Search strategy and sources

The search strategy consisted of two parts. Firstly, PubMed, Web of Science and Embase were searched from inception until 17th of February 2020 to identify all relevant publications reporting data on the association of oral antibiotics with CRC risk. The search string was initially optimised with the help of Karolinska Institutet University Library (Supplementary Table 1), combining Medical Subject Headings (MeSH-terms) and keywords. Secondly, we conducted a comprehensive manual search by screening reference and citation lists of full-text assessed publications, reviews and editorials, and the Cochrane Central Register of Controlled Trials database (https://www.cochranelibrary.com/central). No restrictions were set for the search.

### Study selection and eligibility criteria

All data were exported to EndNote X7 and one (JS) author completed the selection based on publication titles. Two independent authors (J.S. and N.B.) selected the publications by assessing the abstracts and full-texts, and any disagreements were solved by mutual consideration between both authors. One author (J.S.) extracted summary data from the publications and another author (R.F.) crosschecked the extracted risk estimates. To be eligible for this meta-analysis, all following criteria had to be fulfilled:

1. Study providing original data comparing individuals who ever-used (i.e., ever-users) antibiotics with nonusers of antibiotics (0–1 prescriptions), and the risk of primary CRC, colon or rectal cancer.
2. Cohort study, case–control study or randomised controlled trial (RCT).
3. Standardised risk estimates were presented as relative risks (RRs), odds ratios (ORs) or hazard ratios (HRs) with corresponding 95% confidence intervals (CI), or sufficient data were available to calculate these.

Following publications were excluded:

1. Animal and in-vitro studies.
2. Study design: cross-sectional studies, case-reports, abstracts, and reviews, etc.
3. Only pediatric population.
4. Irrelevant exposure or outcome.

In case of publications with overlapping data, the latest publication was included. If the publication year was the same, the most comprehensive study was included.

### Quality assessment

Two independent authors (J.S. and R.F.) completed the quality assessment of the included studies, by systematically applying two quality assessment tools. Newcastle-Ottawa Scale (NOS) tool for cohort and case–control studies^24^ had scores ranging between 0 and 9, with a total score of respectively, ≤3, 4–6 and ≥7 considered indicative of low, moderate and high quality. Additionally, a customised tool for assessing quality and susceptibility to bias in observational studies was applied.^25^ Quality assessment was not used to exclude studies.

### Data items

For each eligible study, we extracted at minimum the following data: (1) study characteristics (i.e. author, year, country, study setting and design, follow-up, risk-estimates, factors adjusted for in the statistical analyses, funding and conflict of interest related information), and (2) population characteristic (i.e. age, anatomical location of CRC, and indication, type and dose of antibiotic use).

### Data synthesis and statistical analysis

We used DerSimonian & Laird random-effects meta-analysis to pool together the most adjusted risk estimates,^26^ providing pooled effect sizes (ES) with 95% CIs for each outcome. Subgroup analyses were performed by anatomical location, study design, antibiotic class and antibiotic group (if reported in two or more studies). Standardised risk estimates (i.e. ORs, RRs and HRs) were considered equivalent, as the outcome is rare. The included antibiotic classes were categorised as follows: penicillins, tetracyclines, sulfonamides, macrolides and lincosamides, quinolones, nitrofurans, cephalosporins, carbapenems, nitroimidazole and metronidazole, imidazole and others (based on reported data). Of these, penicillins, metronidazole and lincosamides were considered narrow-spectrum antibiotics, and the remaining broad-spectrum antibiotics.

We performed dose–response analyses to assess the relationship between the number of days exposed to any antibiotics/number of prescriptions and CRC risk. For analyses on the number of prescriptions, non-users were defined as having received 0–1 prescriptions (based on the available data). This could potentially dilute the estimates towards null. We used the median of each prescription category (e.g. 15 prescriptions for category of 10–20 prescriptions) to quantify these as a continuous variable. Firstly, the dose–response model was fitted within each study and variance-weighted generalised least squares (GLS) regression models were used to estimate the pooled study-specific trends.^27^ Departure from linearity was evaluated by Wald test, with a cut off of *p* < 0.05 indicative of non-linearity. We fitted models with cubic splines and a multivariable random-effects model was used to pool the study-specific estimates.^27^ To select the best model fit, Akaike information criterion (AIC) was applied.^26^ The I^2^ statistics was used to assess statistical across-studies heterogeneity.^28^ Publication bias and small-study effects^26^ were assessed using Egger’s test^29^ and funnel plots.^30^

To evaluate the robustness of our results several sensitivity analyses were undertaken by: (i) excluding a study with selected population,^31^ (ii) excluding studies without a clear lag-time of at least 1-year, (iii) broad- and narrow-spectrum antibiotics, (iv) excluding/restricting to studies adjusting for non-steroidal anti-inflammatory drug (NSAID), (v) restricting to studies excluding patients with inflammatory bowel disease (IBD), and (vi) restricting to studies excluding patients with Crohn’s disease and ulcerative colitis. All statistical analyses were performed with STATA MP 15.1.

## Results

### Included studies

**Fig. 1:**
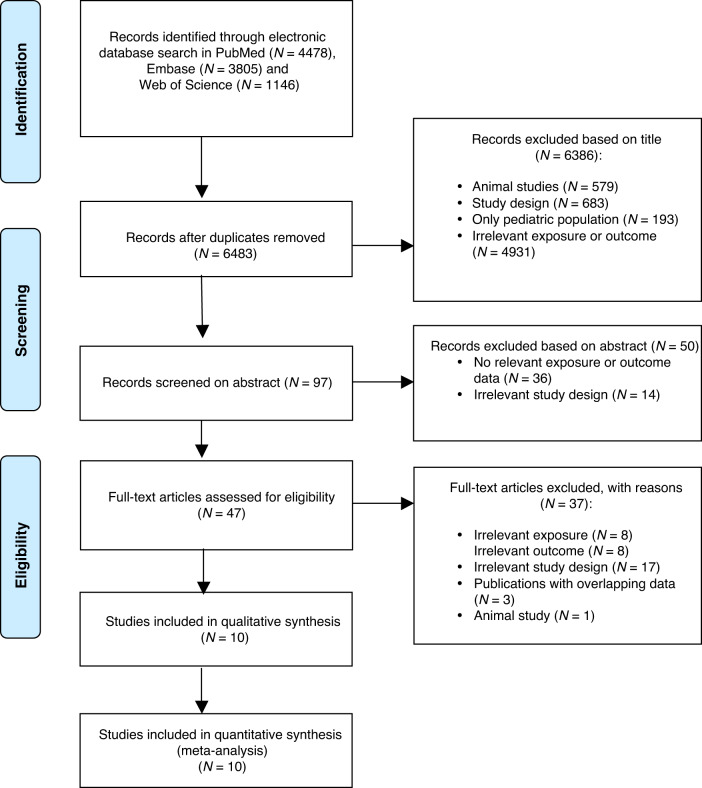
Study selection. A PRISMA Flowchart of the selection of relevant publications included to this meta-analysis.

The search identified 6483 publications, and after the selection process ten studies met the eligibility criteria, including 4,147,560 individuals and over 73,550 CRC cases (Fig. 1).^9,31–39^ Supplementary Table 2 presents all excluded, full-text assessed publications.

Supplementary Table 3 presents the outlining characteristics of the ten included studies. Two were cohort studies^36,37^ and eight were case–control studies,^9,31–35,38,39^ all published in English between 1998 and 2020. Three studies were conducted in the United States (*N* = 3),^32,33,37^ five in Europe (UK *N* = 3,^9,35,39^ Netherlands *N* = 1,^34^ Finland *N* = 1^36^), one in New Zealand^38^, and one in Taiwan.^31^ All publications addressed exposure to oral antibiotics only.^9,31–39^ Six studies^9,31,34,36,38,39^ provided data for cumulative use (i.e. all antibiotic classes combined), and all but two studies^9,36^ provided antibiotic class-specific data (for at least 1 antibiotic class). Five studies provided the number of prescriptions,^9,31,34,35,39^ four reported the number of days exposed,^31,34–36^ and one study utilised cumulative dose expressed as tertiles.^31^ Six studies investigated the risk of primary CRC,^9,34,35,37–39^ and five studies provided data for colon cancer^9,31–33,36^ and three studies for rectal cancer.^9,31,36^ All but three studies reported a clear lag-time of at least 1-year.^9,31,32^ The median time elapsed between first antibiotic prescription and cancer diagnosis was 8 years, ranging between 1 and 15 years.

### Quality assessment

The quality of the included publications was considered high (Supplementary Table 4). In all studies but one,^33^ drug registries were used for exposure ascertainment, enabling prospective data collection and eliminating possible recall bias. The outcome was identified from registries or clinical databases in all studies. Although only two studies reported complete coverage of the region (i.e. >90% of eligible participants),^31,36^ all studies were population-based and adjusted for age. Apart from two publications,^33,35^ the studies clearly excluded antibiotic prescriptions within one year before cancer diagnosis. Additionally, six studies clearly excluded patients with predisposing conditions such as IBD^9,34,35,39^ or Crohn’s disease and ulcerative colitis.^31,33^

### Antibiotics and risk of colorectal, colon and rectal cancer

**Fig. 2:**
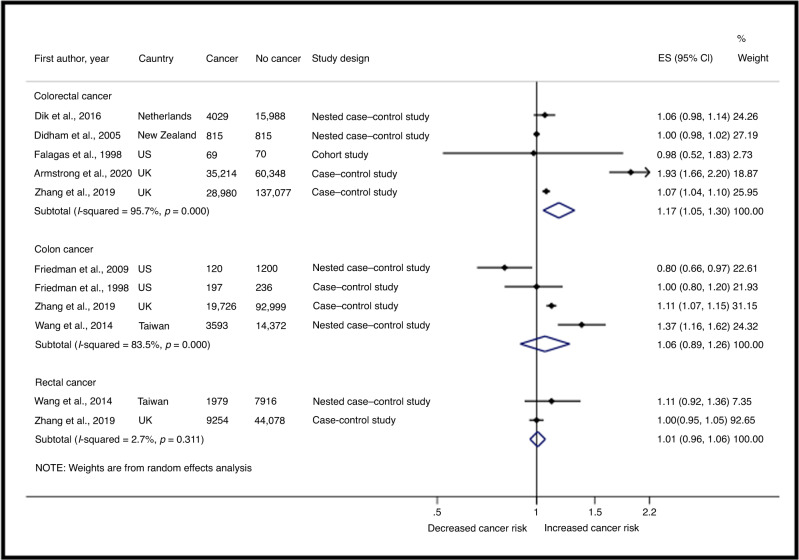
Forest plot of the most adjusted relative risks for the association of oral antibiotic use with colorectal cancer risk, ever-users compared to non-users. ES effect size, 95% CI 95% confidence interval.

For the analysis of antibiotic ever-use versus non-use, eight publications were included (Fig. 2).^9,31–34,37–39^ The pooled risk estimate revealed an increased risk of CRC (effect size ES = 1.17, 95%CI 1.05–1.30, *N* = 5),^9,34,37–39^ whilst no clear association were shown for colon (ES = 1.06, 95%CI 0.89–1.26, *N* = 4)^9,31–33^ and rectal cancer (ES = 1.01, 95%CI 0.96–1.06, *N* = 2),^9,31^ based on fewer studies. The statistical heterogeneity was high for CRC (I^2^ = 95.7%) and colon cancer (I^2^ = 83.5%), but low for rectal cancer (I^2^ = 2.7%). Pooling of the four case–control studies yielded similar effect sizes (ES = 1.17, 95%CI 1.05–1.31, I^2^ = 83.5, *N* = 4) (Supplementary Table 5).^9,34,38,39^ The funnel plot was visually asymmetrical towards positive associations, suggesting for presence of small-study effect (Egger’s test *p* < 0.0005) (Supplementary Fig. 1a). The “missing” studies appeared to be in the area of limited statistical significance (≥90% CI) (Supplementary Fig. 1b).

The sensitivity analysis restricted to studies excluding patients with IBD suggested for a potentially higher CRC risk (ES = 1.28, 95%CI 1.01–1.63, *N* = 3),^9,34,39^ whilst no apparent association was shown for studies excluding patients with Crohn’s disease and ulcerative colitis (ES = 1.16, 95%CI 0.96–1.40, *N* = 2)^31,33^ (Supplementary Table 5). The association with colon cancer remained unchanged after removing the publications without a clearly reported lag-time of at least 1-year.^9,31,32^ Based on fewer studies, the risk of CRC was seemingly lower in studies adjusting for NSAID use (ES = 1.06, 95%CI 1.02–1.11, *N* = 2),^9,34^ compared those not adjusting for NSAID use (ES = 1.26, 95%CI 1.10–1.43, *N* = 6).^31–33,37–39^

### Antibiotic class-specific risk of colorectal cancer

**Fig. 3:**
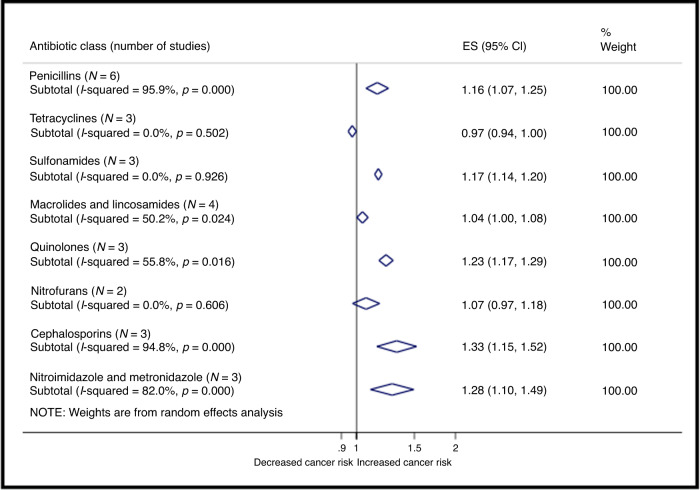
Forest plot for the association of antibiotic use with colorectal cancer, stratified by the different antibiotic classes. Antibiotic ever-users were compared to non-users. ES pooled effect size, 95% CI 95% confidence interval.

The various antibiotic classes (reported in eight publications)^31–35,37–39^ showed different CRC risk estimates (Fig. 3). Compared with non-users, the pooled risk for ever-users was increased for penicillins (ES = 1.16, 95%CI 1.07–1.25, *N* = 6),^31,33–35,38,39^ sulfonamides (ES = 1.17, 95%CI 1.14–1.20, *N* = 3),^34,35,38^ quinolones (ES = 1.23, 95%CI 1.17–1.29, *N* = 3),^34,35,39^ cephalosporins (ES = 1.33, 95%CI 1.15–1.52, *N* = 3)^31,35,37^ and nitroimidazole and metronidazole (ES = 1.28, 95%CI 1.10–1.49, *N* = 3)^32,35,37^ (Supplementary Table 5). A marginally increased risk was found for macrolides and lincosamides (ES = 1.04, 95%CI 1.00–1.08, *N* = 4).^31,34,35,38^ No apparent association was found for other antibiotic classes. Supplementary Table 6 presents the study-specific estimates and meta-analytic weights for each study.

#### Broad- and narrow-spectrum antibiotics

The pooling of broad-spectrum antibiotics revealed an increased CRC risk (ES = 1.70, 95%CI 1.26–2.30, *N* = 3)^31,34,39^ among ever-users, whilst no apparent association with was shown for narrow-spectrum antibiotics (ES = 1.11, 95%CI 0.93–1.32, *N* = 5)^31–33,37,39^ (Supplementary Table 5).

### Dose–response analysis

**Fig. 4:**
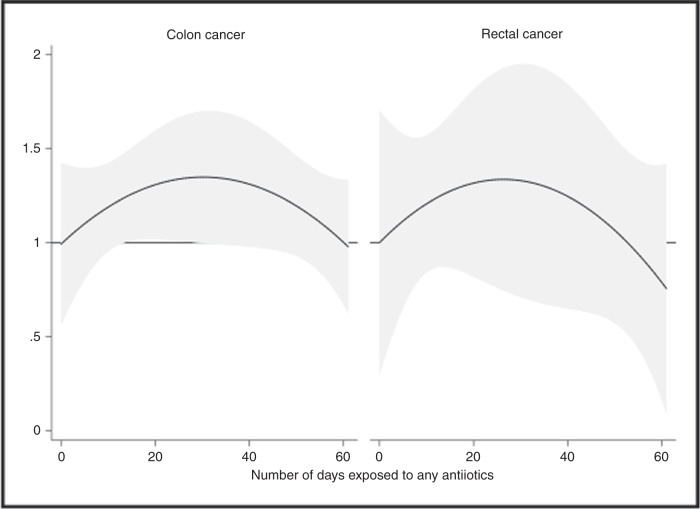
Dose–response relationship of any antibiotic use with colon and rectal cancer, including detection of non-linearity (*p* for non-linearity <0.0005). Data for the number days exposed to any antibiotics were modelled including 3-knot cubic splines, and Akaike Information Criteria was used for best model fit. The reference line represents antibiotic non-users (RR = 1.00) and the vertical axis the risk estimates. The solid line represents the nonlinear trend, and the grey area represents the 95% confidence intervals. RR relative risk, 95%Cl 95% confidence interval.

Five publications reported the number of prescriptions^31,34–36,39^ and four the number of days exposed to antibiotics^9,31,34,35^ (Supplementary Table 5.) The risk of colon and rectal cancer increased with lowest exposure to any antibiotics, following a non-linear risk pattern (*p* for non-linearity <0.0005). The risk appeared to plateau after 30 days of antibiotic use (Fig. 4). The risk patterns were similar for both colon and rectal cancer, with a stabilised risk after high cumulative exposure to antibiotics.

## Discussion

This largest systematic review and dose–response meta-analysis to date provides evidence for antibiotic use being associated with an excess CRC risk, yet the association seemingly differs between antibiotic classes, and possibly by anatomical location of the tumour. Furthermore, this study did not find strong evidence of any particular dose–response association, and the dose–response patterns were similar for colon and rectal cancer with a stabilised risk after high cumulative exposure to any antibiotics.

The major advantage of this study is the inclusion of over 4.1 million individuals and over 73,550 primary CRC cases, enhancing precision compared to previous meta-analyses or single studies.^9,16,21,22^ We excluded anal cancers, because they are commonly originated from squamous cells compared to the epithelial origin of CRC. In sensitivity analyses, we excluded patients with IBD, and the data suggested for a potentially stronger association, yet the inclusion/exclusion of a study^39^ reporting a more extreme risk estimate influenced the pooled risk estimates. In all but one study, the exposure and outcome data were ascertained from registries, eliminating the risk of recall bias.^40^ The publications came from developed countries, including North America, Europe, New Zealand, and Taiwan. The Taiwanese study was based on type II diabetic patients, which may unequivocally introduce some selection, and the lifestyle of this population could differ from the rest of the countries included into this study (due to Westernisation).^31^ Overall, the quality of included the publications was high, based on the Newcastle-Ottawa Scale and a customised quality assessment tool.

The exposure data were mainly restricted to outpatient care drugs, excluding intravenous antibiotics, as their impact on gut microbiota may differ. However, underestimation of exposure due to over-the-counter drug use is unlikely in highly developed countries, where systemic antibiotics are available only on prescription.^41^ Because of the complexity of the exposure, clinical and statistical heterogeneity were acceptable, yet the sensitivity analyses yielded consistent results.

As for all meta-analyses on observational studies, the main limitations of this study are the inability to infer causality and the available data and methods of the included studies. However, to investigate this association by means of a RCT would be challenging, given a large number of individuals should be followed-up over longer time period—limiting feasibility. To see the effects based on real life data, population-based studies offer a favourable and beneficial approach, yet requiring solid research methods and valid data-collection. Another general concern in observational studies is potential residual confounding, and we lacked data to thoroughly assess confounding by indication. Thus, we cannot exclude the possibility that some individuals may have received antibiotics for cancer predisposing conditions, such as impaired immune and inflammation responses,^42,43^ or for as of yet undiagnosed CRC.^44^ To minimise potential reverse causality, sensitivity analyses were restricted to publications clearly reporting at least 1-year lag-time,^33,35^ and the association remained unchanged.

Data were insufficient to compare the risk of left- and right-sided colon cancer, or to evaluate the effect of indication, age or menopausal status. Some evidence suggests for left sided-colon cancer location, particularly in individuals younger than 50 years.^4,9^ This potential association deserves more attention and should be confirmed in other large cohort studies.

Another limitation is that the pooled risk estimates in this study are a mixture of exposures and exposures periods. Despite that, the majority of the studies had rather similar exposure periods (starting from the 1990’s), the patterns of use, formulations and dosages of antibiotics may have changed over time: related to improved global antibiotic stewardship. Additionally, the duration of exposure periods varied between the studies, complicating the interpretation of the pooled risk estimates. Furthermore, considering the high antibiotic consumption, it is possible that non-users have received antibiotics during a lifetime (i.e. before the study period). However, this left censoring and potential misclassification of exposure would likely be at random among antibiotic users and non-users, potentially diluting the risk estimates towards null, rather than explaining the shown increased CRC risk found for some antibiotic classes.

A risk of detection bias is a concern. Antibiotic users could utilise the healthcare services more frequently, and it is possible that antibiotic users could be more likely to participate in potential screening programmes than non-users. This could result in an earlier detection of colon adenomas, thus significantly reducing the risk of developing CRC.^45,46^ It could also lead to an earlier detection of CRC, selectively among antibiotic users,^42,47^ overestimating the CRC risk. However, only two of the included studies adjusted for screening colonoscopy.^9,35^

Compared to previous meta-analyses,^16,21,22^ this present study is the largest and the first to provide evidence for antibiotic-spectrum specific risk differences, and to investigate non-linear dose–response relationship. We also excluded colon adenomas and divided the colorectal continuum into colon and rectal cancer. However, the association between antibiotics and CRC risk is complex, and this study cannot assess the mechanisms underlying the shown risk differences between the various antibiotic classes. Considering the different mechanisms of action, one could expect differences between the various antibiotic classes, and e.g. quinolones are notorious for DNA-damage. Yet, the microbial dysbiosis observed among CRC patients in some studies,^48^ supports the biological plausibility of broad-spectrum antibiotics having a long-term effect on the gut microbiota, potentially facilitating colonisation with pathogenic bacteria to a greater extent than narrow-spectrum antibiotics.^43,48–50^ Emerging amount of evidence from human and animal models suggests that dysbiosis, and particularly some bacteria strains (e.g. *Bacterioides fragilis*, *Escherichia coli* and *Fusobacterium nucleatum*), might involve a higher CRC risk through mechanisms increasing cancer promotion by altering cell proliferation, differentiation and apoptosis, inflammation, and production of DNA damaging toxins.^6,8,48,51,52^ However, if infection and inflammation can independently promote CRC it is possible that a prolonged exposure to antibiotics could reduce CRC risk, by reducing especially chronic inflammation.^18^ This could be one possible explanation, or a contributing factor, for the weak non-linear risk pattern shown in this study. However, a prolonged exposure, particularly for indications requiring high exposure to antibiotics, could indicate for a chronic underlying disease. Thus, confounding by indication cannot be excluded, and since fewer individuals are likely to be exposed to high cumulative dosages than to lower dosages, the dose–response data is likely more reliable for those with lower dosages. Antibiotics have previously been associated with an increased risk of several cancer types, including breast^36,53^ and lung cancer,^16,42^ yet with a decreased risk of ovarian and cervical cancer.^16,54^ Although the risk of confounding by indication cannot be ruled out, it may be more of a concern for lung cancer, considering the high use of antibiotics for respiratory tract infections.^16,42^ Yet, we lacked data on tumour stage and some of the analyses were limited to fewer studies. Thus, our results should be interpreted cautiously.

In conclusion, this systematic review and dose–response meta-analysis suggest that different antibiotic types involve a different CRC risk. In specific, the excess risk was associated with broad-spectrum antibiotics, whilst no apparent association was found for narrow-spectrum antibiotics. The dose–response analysis did not provide strong evidence of any particular dose–response association, and the risk patterns for colon and rectal cancer were similar. Whereas this study cannot determine causality, the findings raise questions highlighting the need for more mechanistic studies on drug–microbiome interactions, and better understanding of the possible role of antibiotics and microbiome in CRC development.

## Supplementary information

Supplementary Materials

## Data Availability

All relevant data is available in this study.
