# Supplementary material for: Antibiotic use and risk of colorectal cancer: a systematic review and dose–response meta-analysis
Source: Br J Cancer. 2020 Sep 24;123(12):1825–32. doi: 10.1038/s41416-020-01082-2 (PMC7722751; doi:10.1038/s41416-020-01082-2)
Supplement: Supplementary file 1 — Supplementary Materials [file 41416_2020_1082_MOESM1_ESM.docx]

**LIST OF SUPPLEMENTARY MATERIALS**

- **Supplementary Table 1**: The Search Strategy
- **Supplementary Table 2**: List of excluded, full-text assessed publications (with a reason for exclusion from this meta-analysis)
- **Supplementary Table 3**: Descriptive characteristics of the publications included in the meta-analysis
- **Supplementary Table 4A & 4B**: Quality assessment of the included publications based two different quality assessment tools for observational studies
- **Supplementary Table 5**: Results of the meta-analysis on association between antibiotics and colorectal cancer
- **Supplementary Figure 1A & 1B**: Funnel plot illustration of publication bias in meta-analysis included studies
- **Supplementary Table 6**: Study specific risk estimates and meta-analytic weights the association of different oral antibiotic classes with colorectal cancer risks.

**Supplementary Table 1: The electronic search strategy**

| **Database** | **Search string** | **Start date** | **Last date of search** | **Number of hits** |
| --- | --- | --- | --- | --- |
| PubMed | (((Anti-bacterial agents [MeSH Term] OR Antibiotic*[Title/Abstract]) OR Antibiotic drug*[Title/Abstract]) AND (("Colorectal Neoplasms"[MeSH Terms] OR colorectal cancer OR colon cancer OR rectal cancer OR neoplasm [Title/Abstract] OR risk factor [Title] OR gastrointestinal [Title]) | Unbound | February 17, 2020 | 4 470 |
| Web of Science | ("Anti-bacterial Agents"[Mesh] OR “antibiotic*” OR “antibiotic drug*”) AND ("Colorectal Neoplasms"[Mesh] OR “colorectal cancer” OR “colon cancer” OR ”rectal cancer” OR “neoplasm”) | Unbound | February 17, 2020 | 1 143 |
| Embase | (‘Antibacterial agents’: ab, ti OR ‘antibiotic*’: ab, ti OR ‘antibiotic drug*’: ab ti) AND (‘colorectal neoplasms’: ab, ti OR ‘colorectal cancer’: ab, ti OR ‘colon cancer’: ab, ti OR ‘rectal cancer’: ab, ti OR ‘risk factor’: ti OR ‘gastrointestinal’: ti OR ‘neoplasms’: ab, ti) | Unbound | February 17, 2020 | 3 769 |

**Supplementary Table 2: All 37 excluded, full-text assessed publications with a reason for exclusion from the meta-analysis.**

| **First author** | **Year** | **Publication title** | **Reason for exclusion** |
| --- | --- | --- | --- |
| Humphrey | 1963 | Clinical experience with the methyl ester of stretoningrin (NCS-45384) | Study design: report |
| Sakakura | 1964 | Clinical trial of endoxan and toyomycin in the field of otorhinolaryngology | Outcome: otorhinolaryngology |
| Goldman | 1980 | Metronidazole: proven benefits and potential risks | Study design: review article |
| Geisbe | 1984 | Colon- und rectumcarcinom | Outcome: operation-accompanying measures |
| Majima | 1987 | Clinical studies of aclacinomycin A (ACM) | Outcome: upper gastrointestinal tract |
| Slattery | 1997 | Energy balance and colon cancer - beyond physical activity | Outcome: energy balance |
| Galandiuk | 2004 | Mutual reporting of process and outcomes enhances quality outcomes for colon and rectal resections | Outcome: colorectal resections |
| Lyman | 2004 | Use of antibiotics and risk of cancer | Study design: letter |
| Stamp | 2004 | Antibiotic therapy may include cancers in the colon and breasts trough a mechanism involving bile acids and colonic bacteria | Study design: correspondence |
| Heymann | 2005 | Antibiotic issues in dermatology | Study design: commentary |
| Kuijsten | 2006 | Plasma enterolignas are associated with lower colorectal adenoma risk | Outcome: colorectal adenoma |
| Aupee | 2009 | La Doxycycline - Medecine tropicale: revue du corps de sante colonial | Study design: review article |
| Sobhani | 2011 | Microbial dysbiosis in colorectal cancer (CRC) patients | Exposure: intestinal microbiota |
| Greer | 2011 | Microbial induction of immunity, inflammation, and cancer | Exposure: gut microbiota |
| Wang | 2012 | Clinical study on risk factor associated with gut flora change in patients with rectal cancer during perioperative period | Outcome: treatment effects on gut flora |
| Ahn | 2013 | Human gut microbiome and risk for colorectal cancer | Exposure: altered community of gut microbes |
| Dinwiddle | 2014 | Recent evidence regarding triclosan and cancer risk | Study design: animal an in-vitro study |
| Francescone | 2014 | Microbiome, inflammation and cancer | Study design: review article |
| Venerito | 2015 | *Helicobacter pylori* and gastrointestinal malignancies | Outcome: gastric cancer |
| Belcheva | 2015 | Gut microbial metabolism and colon cancer: can manipulations of the microbiota be useful in the management of gastrointestinal health? | Exposure: gut microbiota |
| Erdman | 2015 | Gut bacteria and cancer | Exposure: gut microbiota |
| Nanda | 2016 | Doxycycline promotes carcinogenesis & metastasis via chronic inflammatory pathway: an in vivo approach | Population: animal study |
| Tozun | 2016 | Gut microbiome and gastrointestinal cancer: Les liaisons Dangereuses | Study design: review article |
| Printz | 2017 | Long-term antibiotic use associated with cancer-causing polyps | Study design: short communication |
| Journal Nursing Standard | 2017 | Antibiotics linked to heightened risk of bowel cancer precursor | Outcome: bowel cancer precursors |
| Bullman | 2017 | Analysis of *Fucobacterium* persistence and antibiotic response in colorectal cancer | Exposure: *Fusobacterium* and its associated microbiome |
| Adil | 2018 | Association of metronidazole with cancer: a potential risk factor of inconsistent deductions? | Outcome: not CRC |
| Cao | 2018 | Long-term use of antibiotics and risk of colorectal adenoma | Outcome: colorectal adenoma |
| Sanyaolu | 2019 | Antibiotic exposure and the risk of colorectal adenoma and carcinoma: a systematic review and meta-analysis of observational studies | Overlapping data: meta-analysis |
| Bao | 2019 | Association between anti-bacterial drug use and digestive system neoplasms: a systematic review and meta-analysis | Overlapping data: meta-analysis |
| Petrelli | 2019 | Use of antibiotics and risk of cancer: a systematic review and meta-analysis of observational studies | Overlapping data: meta-analysis |
| Mauri | 2019 | Early-onset colorectal cancer in young individuals | Study design: review article |
| Saus | 2019 | Microbiome and colorectal cancer: roles in carcinogenesis and clinical potential | Exposure: gut microbiome |
| Fedirko | 2019 | Antibiotic use and colorectal cancer recurrence and mortality: a Danish nationwide prospective cohort | Study design: conference abstract |
| Lu | 2019 | Association of antibiotic exposure with the mortality in metastatic colorectal cancer patients treated with bevacizumab-containing chemotherapy: a hospital-based retrospective cohort study | Outcome: metastatic colorectal cancer |
| Lavelle | 2019 | Gut microbiome in health and disease: emerging diagnostic opportunities | Exposure: gut microbiome |
| Kester | 2020 | C. Difficile-associated antibiotics alter human mucosal barrier functions by microbiome-independent mechanisms | Outcome: human mucosal barrier functions |

In summary: one of the excluded publications was an animal study, 12 were excluded due to study design, and 21 studies reported irrelevant exposure or outcome data. Additionally, three publications were excluded due to overlapping data.

**Supplementary Table 3: Characteristics of all included studies.**

| **CASE-CONTROL STUDIES** | | | | | | | | | | | |
| --- | --- | --- | --- | --- | --- | --- | --- | --- | --- | --- | --- |
| **First author, year** | **Country** | **Study design** | **Diagnosis period (Exposu-**  **re period)** | **Source of cases** | **Source of controls** | **Number of cases/**  **controls (cohort size)** | **Antibiotic class** | **Measures of dose** | **Age** | **Meno-**  **pausal status** | **The model is conditioned on and adjusted for** |
| Friedman  et al., 1998 | US | Case-control | 1991-1994  (Unclear exposure period) | Individuals aged 30-79 years at primary colon cancer diagnosis, identified from 3 populations (Utah, Minnesota and subscribers of the Kaiser Permanente Medical Care Program in Northern Carolina) | Controls were frequency matched by gender and age, within each population | 197/ 236 (4 403) | Penicillins | Ever-use | Age at diagnosis 30-79 years | Not reported | Gender, age, race, family history of colon cancer, body mass index (BMI), daily intake of calories, fiber and calcium, physical activity, cigarette smoking and alcohol use |
| Didham  et al, 2005 | New Zealand | Nested case-control | 1998-2002  (Maxi-mum 7 years) | Patients diagnosed with primary colorectal cancer (CRC), identified from the Dunedin Royal New Zealand Collage of General Practitioners Research Unit. Data was linked to the New Zealand Hospital Separation Diagnosis database and New Zealand National Mortality database | Matched to cases with 1:1 ratio | 815/ 815  (13 000) | Cumulative use, and macrolides, tetracyclines, cephalosporins, sulfonamides, nitrofurans and others separately | Ever-use | 71 years (±12) | Unclear | Gender, date of birth and active semesters |
| Friedman  et al., 2009 | US | Nested case-control | 1994-2006 (Maxi-  mum 12 years) | Individuals enrolled in the Kaiser Permanente Medical Care Program in Northern Carolina US, diagnosed with primary colon cancer | Randomly selected controls, matched on10:1 ratio to cases by sex, year of birth and year of starting drug coverage | Unclear  (113 278) | Metronidazoles | Ever-use | Unclear | Not reported | Age, sex and year of starting drug coverage in the program |
| Wang  et al., 2014 | Taiwan | Nested case-control | 2000 (Median 7 years) | Type II diabetes patients enrolled in the population-based Taiwan's National Health Insurance, with primary diagnosis of colon or rectal cancer | A risk-set sampling matched on 4:1 ratio to cases, by age, sex and follow-up duration | Colon  3 593/  14 372 and rectum  1 979/  7 916  (640 173) | Cumulative use, and anti-anaerobic/ae-  robic activity, β-lactams, cephalosporins, carbapenems, lincosamides, imidazole and moxifloxacin separately | Ever-use, cumulative dose, number of days exposed and number of prescrip-  tions | Mean age at diagnosis: colon cancer 71 years and rectal cancer 70 years | Not reported | Use of multiple other drugs and comorbidities, number of outpatient visits and hospitalizations |
| Boursi  et al., 2015 | UK | Nested case-control | 1995-2003  (7 years) | All individuals who were 40 years or older at the primary CRC diagnosis, receiving care from the Health Improvement Network practitioner | Selected controls from the database, matched using incidence density sampling on 4:1 ratio to cases by age, sex, practice site and duration of follow-up before index date | 20 990/ 82 054  (103 044) | Penicillins, macrolides, TMP-SMX*, cephalosporins, tetracyclines, quinolones and nitroimidazole separately | Number of days exposed, number of prescrip-tions and timing before CRC diagnosis | Age at diagnosis 71 years (±16) | Not reported | Diabetes mellitus, ischemic heart disease, BMI, smoking, alcohol use, chronic use of Aspirin or non-steroidal anti-inflammatory drugs (NSAIDs) and screening colonoscopy |
| Dik et al., 2016 | Nether-lands | Nested case-control | 2006-2011  (5 years) | Adults (over 18 years) enrolled in the Achmea Health Database in the Netherlands, diagnosed with primary CRC | Randomly selected controls from the database matched on 4:1 ratio to cases, by age and date of birth (and with at least as long follow-up) | 4 029/  15 988  (20 017) | Cumulative use, and penicillins, tetracycline, sulfonamides & trimethoprim, macrolides, quinolones and nitrofurans separately | Ever-use, number of days exposed and number of prescrip-tions | Mean age 71 (±11 years) at diagnosis | 97% of the participants were nonusers of oestrogens. | Date of birth, sex, insulin dependent/ independet diabetes, use of proton pump inhibitors (PPIs), acetylsalisylic acid, NSAIDs, blood lipid-lowering agents, oestrogens, and immunosuppress-sive drugs |
| Zhang  et al., 2019 | UK | Case-control | 1989-2012 (Median 8 years) | Patients between 40-90 years included to the Clinical Practice Research Datalink in UK, diagnosed with primary CRC | Randomly selected controls from the database (without CRC), matched 5:1 ratio to cases, by year of birth, sex, general practitioner (GP) site and database registration year | 28 980/ 137 077 | Cumulative use, and antibiotics with anti-anaerobic and aerobic activity | Ever-use and number of days exposed | Mean age 72 years (unclear when) | Not reported | BMI, smoking, alcohol use, diabetes status, chronic NSAID and aspirin use, and number of colonoscopies |
| Armstrong  et al., 2020 | UK | Case-control | 2008-2018  (Median 6 years) | Patients over 18 years, newly diagnosed with primary CRC, registered in the UK's Royal Collage of General Practitioners' Research and Surveillance Centre | Controls were selected from the database, and matched up to 4:1 to cases on year of birth, gender, index date and the same general practice | 35 214/ 60 348 | Cumulative use, and penicillins and quinolones separately | Ever-use and number of prescrip-tions | Median age 69 years at diagnosis | Not reported | Year of birth, gender, index date and the same general practice, deprivation, ethnicity, smoking, BMI, total cholesterol, coronary heart disease, stroke, chronic obstructive pulmonary disease, liver disease, type 2 diabetes, depression and dementia |
| **COHORT STUDIES** | **Country** | **Study design** | **Follow-up time (exposure period)** | **Definition of the study cohort** | **Ascertainment of exposure and outcome** | **Cohort size** | **Exposures of interest** | **Measures of interest** | **Age** | **Meno-pausal status** | **The model is conditioned on and adjusted for** |
| Falagas  et al., 1998 | US | Matched-cohort study | Median 13 years (exposure period between 1975 and 1983) | Individuals over 18 years enrolled in the Group Health Cooperative (GHC) of Puget Sound, Seattle/US health organization, with >7 years of cancer-free follow-up and diagnosed with primary CRC | Randomly selected non-users matched on 1:1 by age, sex and year of enrollment | 10 444 | Metronidazoles | Ever-use | Median age 40-49 years (as of 1 January 1987) | Not reported | Age, sex and year of GHC enrollment |
| Kilkkinen et al., 2008 | Finland | Cohort study | Maxi-mum 7 years (exposure period between 1995 and 1997) | All individuals between 30-79 years diagnosed with primary colon or rectal cancer, who were residents in Finland on Jan 1st 1995, based on the Population Register | Antibiotic use was ascertained from the Nationwide Drug Prescription Registry. The Finnish Cancer Registry was used to ascertain primary colon and rectal cancers | 3 112 624 | Cumulative use | Number of prescrip-tions | 30-79 years at start of follow-up | 69% of women over 50 years did not receive menopau-sal hormone therapy (MHT) | Age, sex and MHT use |

* TMP-SMX: Trimethoprim/sulfamethoxazole

**Supplementary Table 4A: Quality assessment by means of Newcastle-Ottawa Scale (NOS) tool for case-control and cohort studies.**

| **CASE-CONTROL STUDIES** | **Friedman**  **et al., 1998** | **Didham**  **et al., 2005** | **Friedman**  **et al., 2009** | **Wang**  **et al., 2014** | **Dik**  **et al., 2015** | **Boursi**  **et al., 2015** | **Zhang**  **et al., 2019** | **Armstrong**  **et al., 2020** |
| --- | --- | --- | --- | --- | --- | --- | --- | --- |
| Selection |  |  |  |  |  |  |  |  |
| Is the case definition adequate | 1 | 1 | 1 | 1 | 1 | 1 | 1 | 1 |
| Representativeness of the cases | 0 | 0 | 0 | 0 | 1 | 1 | 1 | 1 |
| Selection of controls | 1 | 1 | 1 | 1 | 1 | 1 | 1 | 1 |
| Definition of controls | 1 | 1 | 1 | 1 | 1 | 1 | 1 | 1 |
| Comparability |  |  |  |  |  |  |  |  |
| Comparability of cases and controls on bases of the design or analysis | 2 | 2 | 2 | 2 | 2 | 2 | 2 | 2 |
| Exposure |  |  |  |  |  |  |  |  |
| Ascertainment of exposure | 0 | 1 | 1 | 1 | 1 | 1 | 1 | 1 |
| Same method of ascertainment for cases and controls | 1 | 1 | 1 | 1 | 1 | 1 | 1 | 1 |
| Non-response rate | 1 | 0 | 0 | 1 | 0 | 0 | 0 | 0 |
| **Total score per study** | **7** | **7** | **7** | **8** | **8** | **8** | **8** | **8** |
| **COHORT STUDIES** | **Falagas**  **et al., 1998** | **Kilkkinen**  **et al., 2008** |  |  |  |  |  |  |
| Selection |  |  |  |  |  |  |  |  |
| Representativeness of the exposed cohort | 0 | 1 |  |  |  |  |  |  |
| Selection of the non-exposed cohort | 1 | 1 |  |  |  |  |  |  |
| Ascertainment of exposure | 1 | 1 |  |  |  |  |  |  |
| Demonstration that outcome of interest was not present at start of study | 1 | 1 |  |  |  |  |  |  |
| Comparability |  |  |  |  |  |  |  |  |
| Comparability of cohorts on the bases of the design or analysis | 2 | 2 |  |  |  |  |  |  |
| Outcome |  |  |  |  |  |  |  |  |
| Assessment of outcome | 1 | 1 |  |  |  |  |  |  |
| Was follow-up long enough for outcomes to occur | 1 | 1 |  |  |  |  |  |  |
| Adequacy of follow-up of cohorts | 1 | 1 |  |  |  |  |  |  |
| **Total score per study** | **8** | **9** |  |  |  |  |  |  |

The scoring ranges from 0-9 scores: selection (0-4 scores), comparability of subjects (0-2 scores), and assessment of exposure and outcome (0-3 scores). A total score of ≤3 indicates for low quality, 4-6 for moderate and ≥7 indicates for high quality.

**Supplementary Table 4B: Quality assessment by means of a customized quality assessment tool.**

**
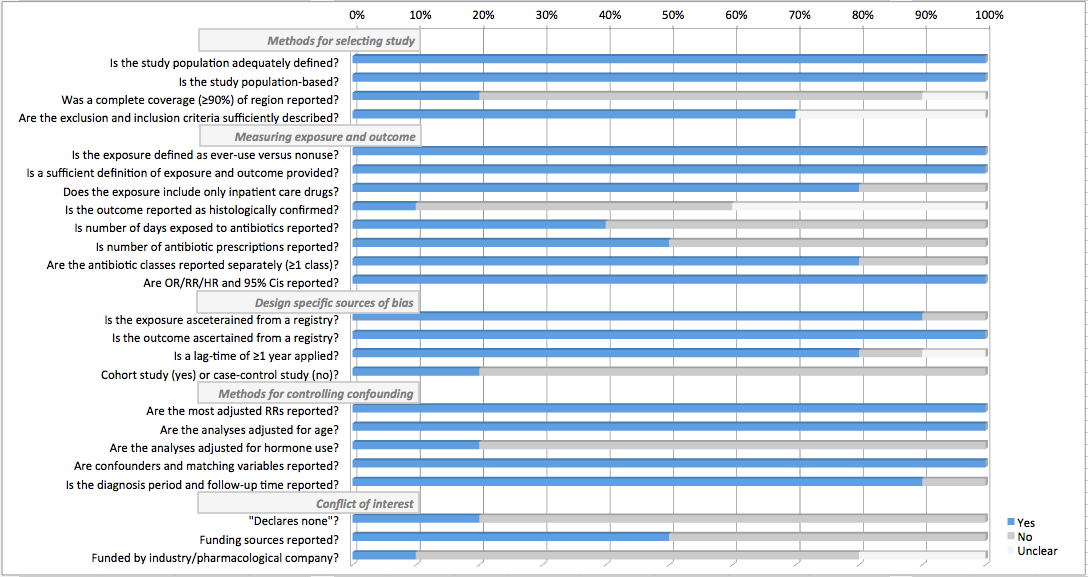
**

The quality variables are presented on the Y-axis, whilst the X-axis shows the percentages of that fulfilled the variables asked for. Thus, 100% means that all of the included studies fulfilled the

criteria, and 0% means that none of the studies included the criteria. Abbreviations: OR: odds ratio, RR: relative risk, HR: hazard ratio, 95%CI: 95% confidence interval.

**Supplementary Table 5: Results of the meta-analysis on the association of oral antibiotic use with risk of colorectal cancer.**

| **All investigated determinants** | **ES** | **95% CI** | **I-squared %** | **Number of studies** | **Reference** |
| --- | --- | --- | --- | --- | --- |
| All antibiotic use | |  |  |  |  |
| *Colorectal cancer* | |  |  |  |  |
| Non-users | 1.00 |  |  |  |  |
| Ever-users | **1**.**17** | **1**.**05-1**.**30** | 95.7 | 5 | 9, 34, 37-39 |
| Study design | |  |  |  |  |
| Cohort studies | NA |  |  | 1 | 37 |
| Case-control studies | **1**.**17** | **1**.**05-1**.**31** | 96.7 | 4 | 9, 34, 38-39 |
| *Colon cancer* | |  |  |  |  |
| Non-users | 1.00 |  |  |  |  |
| Ever-users | 1.06 | 0.89-1.26 | 83.5 | 4 | 9, 31-33 |
| *Rectal cancer* | |  |  |  |  |
| Non-users | 1.00 |  |  |  |  |
| Ever-users | 1.01 | 0.96-1.06 | 2.7 | 2 | 9, 31 |
| Antibiotic class-specific risk estimates for colorectal cancer | | |  |  |  |
| Non-users | 1.00 |  |  |  |  |
| Penicillins | **1**.**16** | **1**.**07-1**.**25** | 95.9 | 6 | 31, 33-35, 38-39 |
| Tetracyclines | 0.97 | 0.94-1.00 | 0.0 | 3 | 34-35, 38 |
| Sulfonamides | **1**.**17** | **1**.**14-1**.**20** | 0.0 | 3 | 34-35, 38 |
| Macrolides and lincosamides | 1.04 | 1.00-1.08 | 50.2 | 4 | 31, 34-35, 38 |
| Quinolones | **1**.**22** | **1**.**17-1**.**29** | 55.8 | 3 | 34-35, 39 |
| Nitrofurans | 1.07 | 0.97-1.18 | 0.0 | 2 | 34, 38 |
| Cephalosporins | **1**.**33** | **1**.**15-1**.**52** | 94.8 | 3 | 31, 35, 37 |
| Nitroimidazole and metronidazole | **1**.**28** | **1**.**10-1**.**49** | 82.0 | 3 | 32, 35, 37 |
| Dose-response analysis by number of prescriptions | | |  |  |  |
| *Colorectal cancer* | |  |  |  |  |
| Dik et al. | **1**.**02** | **1**.**01-1**.**02** |  | 1 | 34 |
| Boursi et al. | **1**.**03** | **1**.**02-1**.**03** |  | 1 | 35 |
| Kilkkinen et al. | 1.01 | 1.00-1.02 |  | 1 | 36 |
| Armstrong et al. | **1**.**07** | **1**.**02-1**.**11** |  | 1 | 39 |
| Pooled dose-response | **1**.**02** | **1**.**01-1**.**04** | 80.5 | 4 | 34-36, 39 |
| *Colon cancer* | |  |  |  |  |
| Wang et al. | 1.00 | 0.98-1.03 |  | 1 | 31 |
| Kilkkinen et al. | **1.02** | **1**.**01-1**.**03** |  | 1 | 36 |
| Pooled dose-response | 1.01 | 1.00-1.02 | 16.9 | 2 | 31, 36 |
| *Rectal cancer* | |  |  |  |  |
| Wang et al. | 1.00 | 0.97-1.03 |  | 1 | 31 |
| Kilkkinen et al. | 1.01 | 0.99-1.02 |  | 1 | 36 |
| Pooled dose-response | 1.00 | 0.99-1.02 | 0.0 | 2 | 31, 36 |
| Dose-response analysis by number of days exposed | | |  |  |  |
| *Colorectal cancer* | |  |  |  |  |
| Dik et al. | 1.00 | 1.00-1.00 |  | 1 | 34 |
| Zhang et al. | 1.00 | 1.00-1.00 |  | 1 | 9 |
| Wang et al. | 1.02 | 0.99-1.05 |  | 1 | 31 |
| Boursi et al. | **1**.**01** | **1**.**01-1**.**01** |  | 1 | 35 |
| Pooled dose-response | 1.00 | 1.00-1.01 | 95.6 | 4 | 9, 31, 34-35 |
| *Colon cancer* | |  |  |  |  |
| Zhang et al. | 1.00 | 1.00-1.00 |  | 1 | 9 |
| Wang et al. | 1.02 | 0.97-1.06 |  | 1 | 31 |
| Pooled dose-response | 1.00 | 1.00-1.00 | 0.0 | 2 | 9, 31 |
| *Rectal cancer* | |  |  |  |  |
| Zhang et al. | 1.00 | 1.00-1.00 |  | 1 | 9 |
| Wang et al. | 1.02 | 0.98-1.06 |  | 1 | 31 |
| Pooled dose-response | 1.00 | 0.99-1.01 | 22.5 | 2 | 9, 31 |
| Sensitivity analyses | |  |  |  |  |
| Excluding a study with selected population | |  |  |  |  |
| *Colon cancer* | |  |  |  |  |
| Non-users | 1.00 |  |  |  |  |
| Ever-users | 0.98 | 0.79-1.19 | 80.8 | 3 | 9, 32-33 |
| Excluding studies without a clear ≥1 year lag-time | | |  |  |  |
| *Colon cancer* | |  |  |  |  |
| Non-users | 1.00 |  |  |  |  |
| Ever-users | 1.08 | 0.86-1.34 | 28.3 | 3 | 9, 31-32 |
| Ever-use of broad-spectrum antibiotics | | |  |  |  |
| *Colorectal cancer* | |  |  |  |  |
| Non-users | 1.00 |  |  |  |  |
| Ever-users | **1**.**70** | **1**.**26-2**.**30** | 99.0 | 3 | 31, 34, 39 |
| Ever-user of narrow-spectrum antibiotics | | |  |  |  |
| *Colorectal cancer* | |  |  |  |  |
| Non-users | 1.00 |  |  |  |  |
| Ever-user | 1.11 | 0.93-1.32 | 93.8 | 5 | 31-33, 37, 39 |
| *Colon cancer* | |  |  |  |  |
| Non-user | 1.00 |  |  |  |  |
| Ever-user | 1.05 | 0.90-1.24 | 81.4 | 3 | 31-33 |
| Studies adjusted for NSAID* use and colorectal cancer risk | | |  |  |  |
| Non-user | 1.00 |  |  |  |  |
| Ever-user | **1**.**06** | **1**.**02-1**.**11** | 72.9 | 2 | 9, 34 |
| Studies not adjusted for NSAID* use and colorectal cancer risk | | | |  |  |
| Non-users | 1.00 |  |  |  |  |
| Ever-users | **1.26** | **1.10-1.43** | 99.1 | 6 | 31-33, 37-39 |
| Studies excluding patients with inflammatory bowel disease and colorectal cancer risk | | | | |  |
| Non-users | 1.00 |  |  |  |  |
| Ever-users | **1**.**28** | **1**.**01-1**.**63** | 96.9 | 3 | 9, 34, 39 |
| Studies excluding patients with Crohn's disease and ulcerative colitis, and colorectal cancer risk | | | | | |
| Non-users | 1.00 |  |  |  |  |
| Ever-users | 1.16 | 0.96-1.40 | 66.7 | 2 | 31, 33 |

Abbreviations: ES: effect size (i.e. relative risk consisting of pooled standardized risk estimates from observational studies), 95% CI: 95% confidence interval, *NSAID: non-steroidal anti-inflammatory drug.

**Supplementary Figures 1A & 1B: Funnel plot illustration of publication bias in meta-analysis included studies examining association of antibiotic ever-use versus nonuse with risk of colorectal cancer.**

Abbreviations: log: logarithm, OR: odds ratio.

**Supplementary Table 6: Study specific risk estimates and meta-analytic weights the association of different oral antibiotic classes with colorectal cancer risks.**

| **Antibiotic class-specific risk estimates for colorectal cancer** | **ES** | **95% CI** | **% Weight*** |
| --- | --- | --- | --- |
| Non-users | 1.00 |  |  |
| *Penicillins* |  |  |  |
| Didham et al. | 1.02 | 0.96-1.07 | 7.89 |
| Dik et al. | 1.14 | 1.04-1.25 | 19.24 |
| Armstrong et al. | 1.49 | 1.45-1.54 | 8.00 |
| Wang et al. | 1.18 | 1.08-1.30 | 13.03 |
| Boursi et al. | 1.13 | 1.10-1.15 | 46.58 |
| Friedman et al. (1998) | 1.00 | 0.82-.22 | 5.26 |
| Pooled risk estimate | 1.16 | 1.07-1.25 | 100.00 |
| *Tetracyclines* |  |  |  |
| Didham et al. | 0.91 | 0.84-0.99 | 12.44 |
| Dik et al. | 1.04 | 0.97-1.12 | 16.19 |
| Boursi et al. | 0.97 | 0.93-1.00 | 71.37 |
| Pooled risk estimate | 0.97 | 0.94-1.00 | 100.00 |
| *Sulfonamides* |  |  |  |
| Didham et al. | 1.12 | 1.01-1.24 | 6.23 |
| Dik et al. | 1.11 | 1.01-1.23 | 6.29 |
| Boursi et al. | 1.17 | 1.14-1.21 | 87.48 |
| Pooled risk estimate | 1.17 | 1.14-1.20 | 100.00 |
| *Macrolides and lincosamides* |  |  |  |
| Didham et al. | 0.95 | 0.87-1.04 | 10.09 |
| Dik et al. | 0.98 | 0.82-1.18 | 10.34 |
| Wang et al. | 1.14 | 1.01-1.28 | 15.63 |
| Boursi et al. | 1.03 | 1.00-1.06 | 63.94 |
| Pooled risk estimate | 1.04 | 1.00-1.08 | 100.00 |
| *Quinolones* |  |  |  |
| Dik et al. | 1.21 | 1.01-1.46 | 18.88 |
| Armstrong et al. | 1.32 | 1.27-1.38 | 20.82 |
| Boursi et al. | 1.23 | 1.19-1.28 | 60.30 |
| Pooled risk estimate | 1.22 | 1.17-1.29 | 100.00 |
| *Nitrofurans* |  |  |  |
| Didham et al. | 0.99 | 0.74-1.33 | 10.47 |
| Dik et al. | 1.08 | 0.98-1.19 | 89.53 |
| Pooled risk estimate | 1.07 | 0.97-1.18 | 100.00 |
| *Cephalosporins* |  |  |  |
| Didham et al. | 0.99 | 0.87-1.12 | 11.38 |
| Wang et al. | 2.41 | 1.85-3.14 | 20.21 |
| Boursi et al. | 1.11 | 0.96-1.28 | 68.40 |
| Pooled risk estimate | 1.33 | 1.15-1.52 | 100.00 |
| *Nitroimidazole and metronidazole* |  |  |  |
| Boursi et al. | 1.46 | 1.40-1.53 | 78.24 |
| Friedman et al. (2009) | 0.80 | 0.66-0.97 | 17.13 |
| Falagas et al. | 0.98 | 0.52-1.84 | 4.64 |
| Pooled risk estimate | 1.28 | 1.10-1.49 | 100.00 |
| Dose-response analysis by number of prescriptions |  |  |  |
| *Colorectal cancer* |  |  |  |
| Dik et al. | 1.02 | 1.01-1.02 | 33.94 |
| Boursi et al. | 1.03 | 1.02-1.03 | 33.21 |
| Kilkkinen et al. | 1.01 | 1.00-1.02 | 27.72 |
| Armstrong et al. | 1.07 | 1.02-1.11 | 5.14 |
| Pooled dose-response | 1.02 | 1.01-1.04 | 100.00 |
| *Colon cancer* |  |  |  |
| Wang et al. | 1.00 | 0.98-1.03 | 23.90 |
| Kilkkinen et al. | 1.02 | 1.01-1.03 | 76.10 |
| Pooled dose-response | 1.01 | 1.00-1.02 | 100.00 |
| *Rectal cancer* |  |  |  |
| Wang et al. | 1.00 | 0.97-1.03 | 22.86 |
| Kilkkinen et al. | 1.01 | 0.99-1.02 | 77.14 |
| Pooled dose-response | 1.00 | 0.99-1.02 | 100.00 |
| Dose-response analysis by number of days exposed |  |  |  |
| *Colorectal cancer* |  |  |  |
| Dik et al. | 1.00 | 1.00-1.00 | 32.25 |
| Zhang et al. | 1.00 | 1.00-1.00 | 32.83 |
| Wang et al. | 1.02 | 0.99-1.05 | 2.97 |
| Boursi et al. | 1.01 | 1.01-1.01 | 31.95 |
| Pooled dose-response | 1.00 | 1.00-1.01 | 95.60 |
| *Colon cancer* |  |  |  |
| Zhang et al. | 1.00 | 1.00-1.00 | 99.94 |
| Wang et al. | 1.02 | 0.97-1.06 | 0.06 |
| Pooled dose-response | 1.00 | 1.00-1.00 | 100.00 |
| *Rectal cancer* |  |  |  |
| Zhang et al. | 1.00 | 1.00-1.00 | 88.75 |
| Wang et al. | 1.02 | 0.98-1.06 | 11.25 |
| Pooled dose-response | 1.00 | 0.99-1.01 | 100.00 |

Abbreviations: ES: effect size (i.e. relative risk consisting of pooled standardized risk estimates from observational studies), 95% CI: 95% confidence interval. *The meta-analytic weights were obtained from a random effects meta-analysis.
